# Complete Genome Sequence of Citricoccus sp. Strain SGAir0253, Isolated from Indoor Air in Singapore

**DOI:** 10.1128/MRA.00606-19

**Published:** 2019-09-12

**Authors:** Lakshmi Chandrasekaran, Daniela I. Drautz-Moses, Akira Uchida, Rikky W. Purbojati, Anthony Wong, Kavita K. Kushwaha, Alexander Putra, Nicolas E. Gaultier, Balakrishnan N. V. Premkrishnan, Cassie E. Heinle, Vineeth Kodengil Vettath, Ana Carolina M. Junqueira, Stephan C. Schuster

**Affiliations:** a Singapore Centre for Environmental Life Sciences Engineering, Nanyang Technological University, Singapore; b Departamento de Genética, Instituto de Biologia, Universidade Federal do Rio de Janeiro, Rio de Janeiro, Brazil; Broad Institute

## Abstract

Citricoccus sp. strain SGAir0253 was isolated from indoor air collected in Singapore. Its genome sequence was assembled using single-molecule real-time sequencing. It comprises one chromosome of 3.32 Mb and two plasmids of 137 kb and 99 kb. The genome consists of 2,950 protein-coding genes, 49 tRNAs, and 9 rRNAs.

## ANNOUNCEMENT

Members of the bacterial genus Citricoccus are Gram-positive, nonmotile bacteria classified in the phylum Actinobacteria, family Micrococcaceae (1). The first reported draft genome sequence of a *Citricoccus* sp. was of an isolate from the Cuatro Cienegas Basin (CCB) in Coahuila, Mexico (2). The members of this genus have been isolated and reported from various environments, such as marine macroalgae (3), mold-colonized walls (4), saline wastewater bioreactors (5), wastewater treatment plants (6), medieval wall paintings (1), desert soil (7), and human skin (8). There are no previous reports indicating the presence of *Citricoccus* spp. in air samples, and therefore, this work provides the first evidence that members of this genus can also be found in air.

The strain SGAir0253 was isolated from an indoor air sample collected in Singapore (global position system coordinates, 1.304842°N, 103.791614°E) using the Andersen single-stage impactor (SKC, USA). The air was impacted onto marine agar (Becton, Dickinson, USA), and further isolation of colonies was carried out by culturing onto Trypticase soy agar (TSA) at 30°C. A single colony was grown in lysogeny broth (Becton, Dickinson) at 30°C overnight and subjected to DNA extraction using the Wizard genomic DNA purification kit (Promega, USA), according to the manufacturer’s protocol. Library preparation was performed with the SMRTbell template prep kit 1.0 (Pacific Biosciences), followed by single-molecule real-time (SMRT) sequencing on the Pacific Biosciences RS II platform.

For the following analysis, default parameters were used for all software unless stated otherwise. Quality control of raw reads was done with PreAssembler filter v1 within the Hierarchical Genome Assembly Process (HGAP) version 3 protocol (9) implemented in the PacBio SMRT Analysis 2.3.0 package. HGAP was also used for the *de novo* assembly of 78,591 subreads, followed by polishing with Quiver (9). The consensus assembly generated three contigs (Table 1). All three contigs were circularized using Circlator 1.1.4 (10).

**TABLE 1:** Assembly statistics for *Citricoccus* sp. strain SGAir0253

Contig	Length (bp)	Coverage (fold)	G+C content (%)	GenBank accession no.
Chromosome	3,323,969	202	73.8	CP039424
Plasmid 1	137,948	221	67.4	CP039425
Plasmid 2	99,421	175	68.4	CP039426

Taxonomic identification was performed using average nucleotide identity (ANI) analysis (11) against a database of bacterial refseq genomes that was created using a text filter for “type, synonymtype, proxytype,” which showed 82% identity with Tepidibacter formicigenes. Phyla-AMPHORA (12) was run using MarkerScanner.pl with an added –DNA flag and MarkerAlignTrim.pl with options –With reference and –outputFormat phylip. Phylotyping.pl was run with default parameters, which showed 89.4% identity to Micrococcus luteus. As the identity was below the threshold for species-level identification for ANI results, we also performed 16S identification by extracting the full-length 16S rRNA gene sequence with Barrnap 0.7 (13). The BLASTn (14) alignment of the 16S rRNA gene sequence to the Silva database resulted in 98.7% identity to *Citricoccus* sp. strain CH26A. Hence, only genus-level assignment was done for this strain.

The NCBI Prokaryotic Genome Annotation Pipeline (PGAP) version 4.2 (15) was used for genome annotation. A total of 3,134 genes were predicted, comprising 2,950 protein-coding genes (PCGs), 9 rRNA operons (3 each of 5S, 16S, and 23S rRNAs), 49 tRNAs, 3 noncoding RNAs, and 123 pseudogenes. Annotation for plasmid 1 predicted proteins carrying antibiotic resistance (class A beta-lactamase, major facilitator superfamily [MFS], and tetracycline [TetR]), as well as protein CopG, which is responsible for copy number regulation of the plasmid. Plasmid 2 contains metal resistance proteins (copper and arsenic). Both plasmids also contain the plasmid mobilization relaxasome protein MobC and a ParA family protein which is essential for plasmid partition.

### Data availability.

The complete genome sequences of *Citricoccus* sp. strain SGAir0253 and its plasmids are available in DDBJ/EMBL/GenBank under accession numbers CP039424, CP039425, and CP039426 and SRA accession number SRR9043820.

## References

[1] Altenburger P, Kampfer P, Schumann P, Steiner R, Lubitz W, Busse H-J 2002 *Citricoccus muralis* gen. nov., sp. nov., a novel actinobacterium isolated from a medieval wall painting. Int J Syst Evol Microbiol 52:2095–2100. doi:10.1099/00207713-52-6-2095. 12508874

[2] Hayano-Kanashiro C, López-Arredondo DL, Cruz-Morales P, Alcaraz L-D, Olmedo G, Barona-Gómez F, Herrera-Estrella L 2011 First draft genome sequence of a strain from the genus *Citricoccus*. J Bacteriol 193:6092–6093. doi:10.1128/JB.06045-11. 21994924 PMC3194908

[3] Leiva S, Alvarado P, Huang Y, Wang J, Garrido I 2015 Diversity of pigmented Gram-positive bacteria associated with marine macroalgae from Antarctica. FEMS Microbiol Lett 362:fnv206. doi:10.1093/femsle/fnv206. 26507390

[4] Schäfer J, Martin K, Kampfer P 2010 *Citricoccus parietis* sp. nov., isolated from a mould-colonized wall and emended description of Citricoccus alkalitolerans Li et al. 2005. Int J Syst Evol Microbiol 60:271–274. doi:10.1099/ijs.0.012567-0. 19651738

[5] Meng F-X, Yang X-C, Yu P-S, Pan J-M, Wang C-S, Xu X-W, Wu M 2010 *Citricoccus zhacaiensis* sp. nov., isolated from a bioreactor for saline wastewater treatment. Int J Syst Evol Microbiol 60:495–499. doi:10.1099/ijs.0.011635-0. 19654363

[6] Nielsen MB, Kjeldsen KU, Ingvorsen K 2011 Description of *Citricoccus nitrophenolicus* sp. nov., a para-nitrophenol degrading actinobacterium isolated from a wastewater treatment plant and emended description of the genus Citricoccus Altenburger et al. 2002. Antonie Van Leeuwenhoek 99:489–499. doi:10.1007/s10482-010-9513-6. 20882410

[7] Li W-J, Chen H-H, Zhang Y-Q, Kim C-J, Park D-J, Lee J-C, Xu L-H, Jiang C-L 2005 *Citricoccus alkalitolerans* sp. nov., a novel actinobacterium isolated from a desert soil in Egypt. Int J Syst Evol Microbiol 55:87–90. doi:10.1099/ijs.0.63237-0. 15653858

[8] Ndiaye C, Bassene H, Cadoret F, Raoult D, Lagier JC, Sokhna C 2018 ‘*Citricoccus massiliensis*’ sp. nov., a new bacterial species isolated from human skin by culturomics. New Microbes New Infect 23:83–85. doi:10.1016/j.nmni.2018.02.004. 29692910 PMC5913060

[9] Chin C-S, Alexander DH, Marks P, Klammer AA, Drake J, Heiner C, Clum A, Copeland A, Huddleston J, Eichler EE, Turner SW, Korlach J 2013 Nonhybrid, finished microbial genome assemblies from long-read SMRT sequencing data. Nat Methods 10:563–569. doi:10.1038/nmeth.2474. 23644548

[10] Hunt M, Silva ND, Otto TD, Parkhill J, Keane JA, Harris SR 2015 Circlator: automated circularization of genome assemblies using long sequencing reads. Genome Biol 16:294. doi:10.1186/s13059-015-0849-0. 26714481 PMC4699355

[11] Shen L, Liu Y, Xu B, Wang N, Zhao H, Liu X, Liu F 2017 Comparative genomic analysis reveals the environmental impacts on two Arcticibacter strains including sixteen Sphingobacteriaceae species. Sci Rep 7:2055. doi:10.1038/s41598-017-02191-4. 28515455 PMC5435697

[12] Wang Z, Wu M 2013 A phylum-level bacterial phylogenetic marker database. Mol Biol Evol 30:1258–1262. doi:10.1093/molbev/mst059. 23519313

[13] Seemann T 2013 Barrnap 0.7: rapid ribosomal RNA prediction.

[14] Altschul SF, Madden TL, Schäffer AA, Zhang J, Zhang Z, Miller W, Lipman DJ 1997 Gapped BLAST and PSI-BLAST: a new generation of protein database search programs. Nucleic Acids Res 25:3389–3402. doi:10.1093/nar/25.17.3389. 9254694 PMC146917

[15] Tatusova T, DiCuccio M, Badretdin A, Chetvernin V, Nawrocki EP, Zaslavsky L, Lomsadze A, Pruitt KD, Borodovsky M, Ostell J 2016 NCBI Prokaryotic Genome Annotation Pipeline. Nucleic Acids Res 44:6614–6624. doi:10.1093/nar/gkw569. 27342282 PMC5001611

